# Purification and Characterization of Recombinant N-Terminally Pyroglutamate-Modified Amyloid-β Variants and Structural Analysis by Solution NMR Spectroscopy

**DOI:** 10.1371/journal.pone.0139710

**Published:** 2015-10-05

**Authors:** Christina Dammers, Lothar Gremer, Philipp Neudecker, Hans-Ulrich Demuth, Melanie Schwarten, Dieter Willbold

**Affiliations:** 1 Institute of Complex Systems (ICS-6) Structural Biochemistry, Forschungszentrum Jülich, 52425 Jülich, Germany; 2 Institut für Physikalische Biologie, Heinrich-Heine-Universität Düsseldorf, 40225 Düsseldorf, Germany; 3 Fraunhofer Institute for Cell Therapy and Immunology, Dep. Molecular Drug Biochemistry and Therapy, 06120 Halle (Saale), Germany; George Washington University, UNITED STATES

## Abstract

Alzheimer’s disease (AD) is the leading cause of dementia in the elderly and is characterized by memory loss and cognitive decline. Pathological hallmark of AD brains are intracellular neurofibrillary tangles and extracellular amyloid plaques. The major component of these plaques is the highly heterogeneous amyloid-β (Aβ) peptide, varying in length and modification. In recent years pyroglutamate-modified amyloid-β (pEAβ) peptides have increasingly moved into the focus since they have been described to be the predominant species of all N-terminally truncated Aβ. Compared to unmodified Aβ, pEAβ is known to show increased hydrophobicity, higher toxicity, faster aggregation and β-sheet stabilization and is more resistant to degradation. Nuclear magnetic resonance (NMR) spectroscopy is a particularly powerful method to investigate the conformations of pEAβ isoforms in solution and to study peptide/ligand interactions for drug development. However, biophysical characterization of pEAβ and comparison to its non-modified variant has so far been seriously hampered by the lack of highly pure recombinant and isotope-enriched protein. Here we present, to our knowledge, for the first time a reproducible protocol for the production of pEAβ from a recombinant precursor expressed in *E*. *coli* in natural isotope abundance as well as in uniformly [*U*-^15^N]- or [*U*-^13^C, ^15^N]-labeled form, with yields of up to 15 mg/l *E*. *coli* culture broth. The chemical state of the purified protein was evaluated by RP-HPLC and formation of pyroglutamate was verified by mass spectroscopy. The recombinant pyroglutamate-modified Aβ peptides showed characteristic sigmoidal aggregation kinetics as monitored by thioflavin-T assays. The quality and quantity of produced pEAβ40 and pEAβ42 allowed us to perform heteronuclear multidimensional NMR spectroscopy in solution and to sequence-specifically assign the backbone resonances under near-physiological conditions. Our results suggest that the presented method will be useful in obtaining cost-effective high-quality recombinant pEAβ40 and pEAβ42 for further physiological and biochemical studies.

## Introduction

Alzheimer's disease (AD) is a neurodegenerative disorder characterized by progressive decline of cognitive functions and has become the main cause for dementia in the elderly [1, 2]. Pathological hallmarks of AD are intracellular neurofibrillary tangles and the accumulation of extracellular amyloid plaques [3, 4]. Amyloid-β (Aβ), the major component of these amyloid plaques, is produced by cleavage of the amyloid precursor protein through β- and γ-secretases, generating various Aβ isoforms varying in length [5–9]. Besides Aβ isoforms starting with the amino acid (aa) D at position 1 (D1), a significant amount of N-terminally truncated Aβ variants is deposited in the brains of AD patients [10, 11], whereby pyroglutamate (pE)-modified Aβ species were described as the major isoforms [12–15]. Up to 20% of the total Aβ are reported to bear a pE residue at the N-terminus [16]. N-terminally truncated pEAβ(3-x) species, with the first two N-terminal aa D1 and A2 being absent, are dominant isoforms in AD brains [17, 18] and are present in up to equivalent amounts compared to full-length Aβ(1-x) in senile plaques [19–21]. The intracellular amount of pEAβ increases with age and it is predominantly found in lysosomes of neurons and neuroglia [22]. pEAβ plays a central role in triggering neurodegeneration and lethal neurological deficits [23, 24]. Thus, N-terminally modified Aβ isoforms represent highly desirable therapeutic targets and became more important in the recent years [15, 25–27].

Aβ(3-x) can be generated by the removal of the first two aa (D1 and A2) from Aβ(1-x) or by alternative splicing, leading to the N-terminal aa E3. The enzyme glutaminyl cyclase (QC) catalyzes intra-E lactam ring formation involving the N-terminal amino group of E3 and its γ-carboxyl group by dehydration leading to pEAβ [28, 29]. Although N-terminal pE formation is a preferred enzymatic reaction [30], it can also be achieved non-enzymatically [31]. This reaction is accelerated with an N-terminal Q residue as a substrate instead of E [32].

The conversion results in altered biophysical and biochemical properties since: (1) pEAβ shows higher hydrophobicity due to the formation of the N-terminal pE lactam ring and the loss of three charges resulting in increased aggregation propensity [12, 19]. (2) The blocked N-terminus leads to higher stability since it is inaccessible for degradation by aminopeptidases. (3) pEAβ shows faster aggregation kinetics with up to 250-fold acceleration and (4) is also more neurotoxic as compared with corresponding non-N-terminally truncated Aβ species independent of their C-terminal lengths [24, 33–36].

A deeper understanding of the molecular mechanisms of pEAβ formation, aggregation and its structure may provide new insights into the difference compared to Aβ and its role in AD. Structural data from NMR spectroscopy could extend the knowledge of pEAβ pathogenicity and will give information about ligand interactions for rational drug design. However, large amounts of pEAβ are needed for such studies. Principally, peptides up to 100 amino acid residues can be prepared chemically by solid phase synthesis, but when it comes to isotope-enriched peptides this strategy becomes very costly and constant biological activity is not guaranteed since there are often differences in purity between the batches [37].

Here, we report a method for reproducible expression and purification of recombinant pEAβ(3–40) and pEAβ(3–42) with natural isotope abundance, as well as uniformly [*U*-^15^N] or [*U*-^13^C, ^15^N]-labeled protein with yields up to 15 mg/l culture based on a previously published protocol for Aβ by Finder, Glockshuber and coworkers [37]. To avoid time and cost consuming enzymatic pE formation by QC, we applied conditions for nonenzymatic pE formation on Aβ(E3Q-x) mutants leading to complete and more rapid pEAβ formation compared to the Aβ(E3-x) species. The chemical and conformational states of the purified pEAβ proteins were characterized biophysically by mass spectrometry, thioflavin-T (ThT) assay and solution NMR spectroscopy.

## Results and Discussion

### Cloning, expression and purification of the mutants Aβ(E3Q-40/42)

The fusion constructs Aβ(E3Q-40) and Aβ(E3Q-42) are based on the recombinant Aβ(1–42) *E*. *coli* derived construct published by Finder, Glockshuber and coworkers [37] consisting of a His_6_-tag, a solubilizing fusion partner (NANP)_19_, established previously [38], followed by a TEV protease recognition and cleavage site and the Aβ sequence 3-40/42. E3 was replaced by Q in order to improve the non-enzymatic reaction to pE (Method A in S1 File). The protease recognition site thus is now modified to ENLYFQ↓Q, where the arrow indicates the cleavage site, leading to Q3 as the N-terminal aa in the resulting Aβ constructs. Typically, the TEV protease recognition and cleavage site contains a G or a S C-terminal of the TEV protease cut, but as proven previously, an exchange of G or S to Q leads to 90% cleavage efficiency [39]. Thus Q becomes the first aa (Q3) of the cleavage product, which is readily susceptible to non-enzymatic pE formation under mild acidic and elevated temperature conditions. To show the applicability and advantage of this mutation for non-enzymatic pEAβ conversion, we additionally produced Aβ(3–42) starting at the N-terminal position with the original E instead of Q, which *in vivo* is the primary substrate for QC and is catalytically converted to pEAβ but can also be modified non-enzymatically [28]. We found that Aβ(3–42) converted to pEAβ significantly slower than Aβ(E3Q-42) with Q at the N-terminal position.

**Fig 1:**
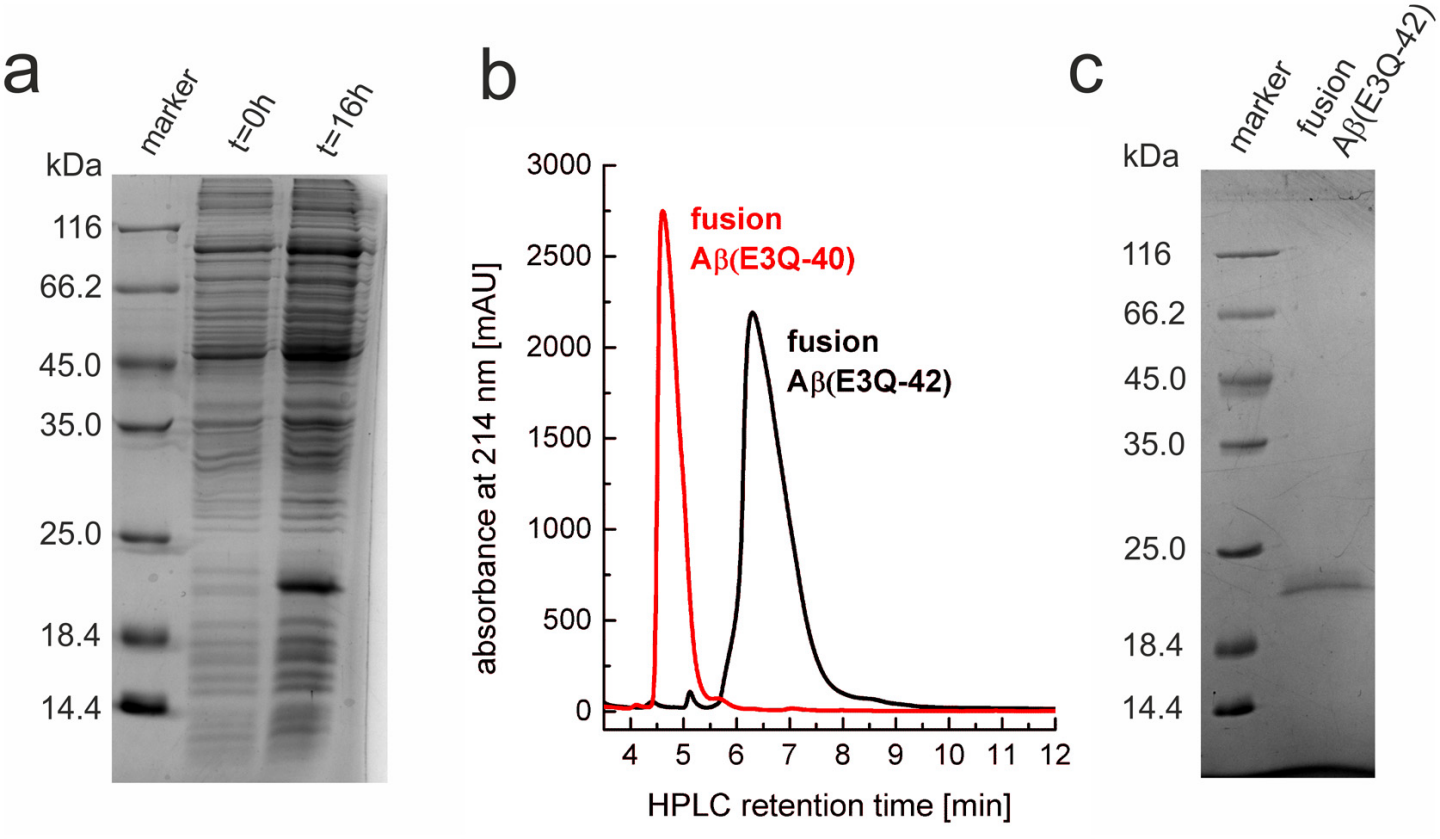
Expression and purification of fusion Aβ(E3Q-42) protein in *E*. *coli* BL21 (DE3) pLysS. (a) 15% Tris/Glycine-SDS-PAGE analysis of the lysates before IPTG induction (t = 0 h) and after IPTG induction leading to expression of fusion Aβ(E3Q-42) (t = 16 h after induction) at 30°C. (b) Analytical RP-HPLC of IMAC purified fusion Aβ(E3Q-40) (red) and fusion Aβ(E3Q-42) (black) with typical retention times of 5 and 7 min, respectively. (c) 15% Tris/Glycine-SDS-PAGE of IMAC and RP-HPLC purified fusion Aβ(E3Q-42).

Expression of the fusion constructs in *E*. *coli* BL21 (DE3) pLysS was obtained at a high cell density of OD_600nm_ ≥ 1.2. Reducing the temperature after induction to 30°C and expression overnight resulted in a large amount of fusion protein accumulated in inclusion bodies (Fig 1a). Denaturing conditions were necessary to solubilize these inclusion bodies. The first purification step was an IMAC in 8 M GdmCl. One-step washing of the IMAC column with 20 mM imidazole and subsequent elution with 500 mM imidazole was performed to isolate the fusion protein and to remove most of non-specifically bound impurities as analyzed by analytical RP-HPLC (Fig 1b). Typical retention time of fusion Aβ(E3Q-40) was 5 min and 7 min for fusion Aβ(E3Q-42). The fusion proteins were further purified using preparative RP-HPLC and lyophilized from aqueous ACN resulting in pure fusion protein determined via SDS-PAGE according to Laemmli [40] (Fig 1c, Method B in S1 File). The following yields per l of cell culture were obtained as shown in Table 1: 200 ± 5 mg for fusion Aβ(E3Q-40/42) and fusion Aβ(3–42) in natural abundance, 25 ± 3 mg for [*U*-^15^N] fusion Aβ(E3Q-40/42) and 20 ± 3 mg for [*U*-^13^C, ^15^N] fusion Aβ(E3Q-40/42).

**Table 1:** Yields of intermediates and final purified pEAβ peptides in mg per l culture broth.

	Natural abundant [mg/l]	[*U*-^15^N] labeled [mg/l]	[*U*-^13^C,^15^N] labeled [mg/l]
fusion Aβ(E3Q-40)	205	28	23
Aβ(E3Q-40)	42	5	4.5
pEAβ40	15	2.3	2
fusion Aβ(E3Q-42)	200	24	20
Aβ(E3Q-42)	41	4.8	4
pEAβ42	14	2.1	1.8

**Fig 2:**
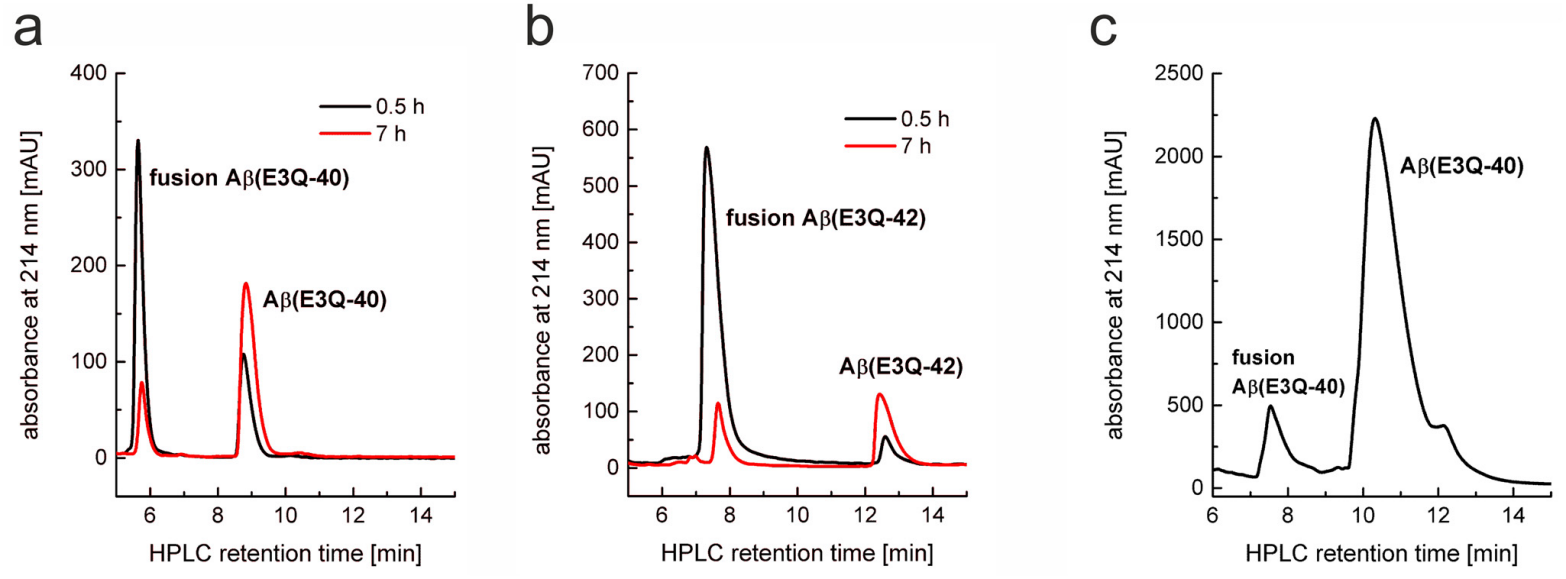
Analytical RP-HPLC of TEV protease cleavage reaction of Aβ(E3Q-40) (a) and Aβ(E3Q-42) (b). The peptides were applied on an analytical Zorbax SB-300 C8 column and eluted with 30% ACN and 0.1% TFA at 80°C. Black lines indicate analysis after 0.5 h and red lines after 7 h TEV cleavage reaction at 4°C. Peaks for the fusion proteins decreased while peaks indicating cleaved Aβ(E3Q-40) or Aβ(E3Q-42) were increasing. (c) Semi-preparative RP-HPLC of TEV-cleaved fusion Aβ(E3Q-40) to separate Aβ(E3Q-40) from remaining fusion protein.

Next, the lyophilized fusion proteins were analyzed for efficient TEV protease cleavage. It turned out, that high molar ratios of TEV protease were necessary to balance the modified cleavage site, i.e. ENLYFQ↓Q, instead of ENLYFQ↓G/S. Enhanced cleavage reaction was achieved by lowering the incubation temperature to 4°C by decreasing the aggregation of the cleaved Aβ with remaining fusion protein (Method C in S1 File). Most of the fusion Aβ(E3Q-40/42) were cleaved within 7 h, as proven by analytical RP-HPLC (Fig 2a and 2b). Retention-time of cleaved Aβ(E3Q-40) was approximately 8.9 min and 12.2 min for Aβ(E3Q-42). After overnight incubation, cleaved Aβ(E3Q-42) and Aβ(3–42) precipitated during the reaction completely, whereas around 50% of Aβ(E3Q-40) stayed in solution. Precipitates were resolved in 8 M GdmCl for further purification. Chromatograms of preparative HPLC indicate that there was still some fusion protein remaining (Fig 2c) which could be removed by adjusting the gradient as described in the Materials and Methods section. Cleaved Aβ was separated from the fusion-tag and TEV protease and lyophilized. As the fusion-tag accounts for 70% of the total fusion protein, a maximum of 30 mg target protein per 100 mg fusion protein is theoretically obtainable with 100% cleavage efficiency. In total, approximately 20 mg purified cleaved Aβ(E3Q-40/42) per 100 mg fusion protein were received.

### Conversion to pEAβ40 and pEAβ42

**Fig 3:**
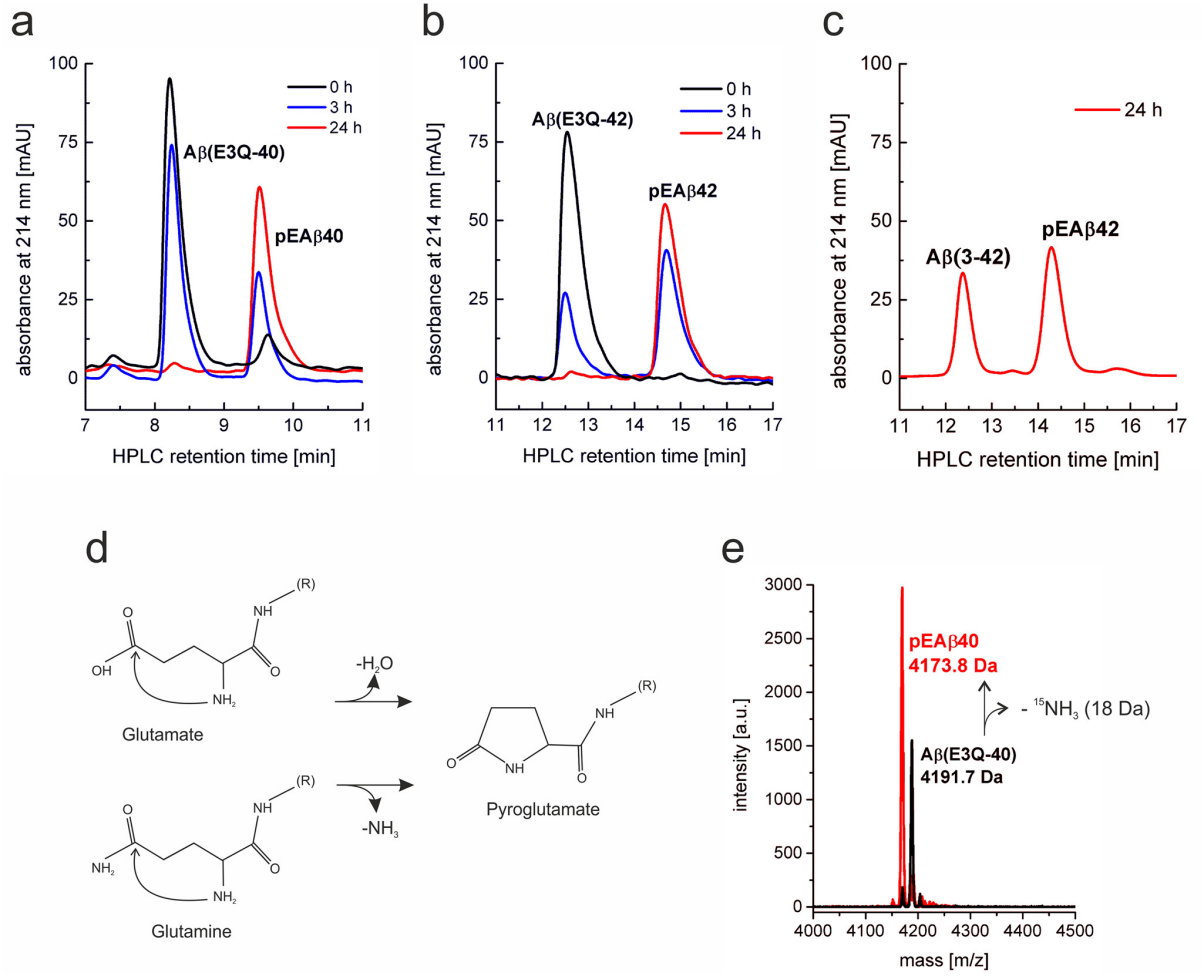
Non-enzymatic pyroglutamate (pE) formation by acidic and elevated temperature conditions of Aβ(E3Q-40) (a), Aβ(E3Q-42) (b) and wild type Aβ(3–40) (c). Peptides were incubated at 45°C in sodium acetate buffer pH 3.5 for 24 h. Conversion was observed by analytical RP-HPLC with an analytical Zorbax SB-300 C8 column in 30% ACN/ 0.1% TFA at 80°C. Peaks for non-modified peptides decreased while peaks for the pEAβ variants appeared at a longer retention time. (d) Reaction scheme of the conversion of N-terminal E3 or N-terminal Q3 to pE. (e) Mass spectrometry of [*U*-^15^N]Aβ(E3Q-40) compared to N-terminally modified [*U*-^15^N]pEAβ40. Molecular mass of the peptides differs in 18 Da due to the loss of the ^15^NH_3_ group.

It is known, that N-terminal pE formation from E is a preferred enzymatical reaction [30], but also can be achieved non-enzymatically under mild acidic conditions and increased temperature [31]. However, both enzymatic and non-enzymatic intra-molecular lactam formation with an N-terminal Q residue instead of E is much faster [32]. For this reason, we decided to use the mutant Aβ(E3Q) for spontaneous pE formation and compared it with a construct bearing E3 at the N-terminus. The reaction schemes for the conversion to pE from N-terminal E by dehydration or from N-terminal Q by subtraction of ammonia are shown in Fig 3d. Purified Aβ(E3Q-40), Aβ(E3Q-42) and Aβ(3–42) were dissolved in acetate buffer at pH 3.5 and incubated at 45°C for spontaneous pE formation (Method D in S1 File). Reaction was observed with analytical RP-HPLC at the start of the reaction, after 3 h and after 24 h incubation, respectively (Fig 3a and 3b). Due to the loss of the positively charged hydrophilic amino group, the pE-modified peptides get more hydrophobic resulting in a longer retention time on RP-HPLC. For Aβ(E3Q-40)and Aβ(E3Q-42), the initial peptide peaks eluting at 8.2 or 12.6 min decreased over time while new peaks eluting at 9.5 or 15 min emerging due to pE conversion of Aβ(E3Q-40) and Aβ(E3Q-42) appeared. MALDI-mass spectrometry (see below) proved the conversion to the corresponding pE-modified variants. An incubation time of 24 h was appropriate to convert most of Aβ(E3Q-40/42) to pEAβ40 and pEAβ42, respectively, as observable by RP-HPLC analytics (Fig 3a and 3b).

Conversion was proven by comparing a non-pE-converted sample of [*U*-^15^N]-Aβ(E3Q-40) with [*U*-^15^N]-pEAβ40 using MALDI-mass spectrometry (Fig 3e, Method F in S1 File). The calculated averaged mass of [*U*-^15^N]-Aβ(E3Q-40) is 4194 Da and 4176 Da for [*U*-^15^N]-pEAβ40. Major peaks differing in 18 Da mass were visible, which corresponds to the loss of ^15^NH_3_. However, the exact mass of both peptides were 2 Da less than calculated based on the fact that the purified protein is not monoisotopic, but at least 95% of all nitrogen atoms are ^15^N isotopes. Non-enzymatic conversion of Aβ(3–42) containing the N-terminal E3 to pEAβ42 showed a pronounced decreased efficiency under exactly the same conditions, i.e. 24 h incubation time at 45°C incubation temperature with sodium acetate, pH 3.5, as buffer condition. Only approximately 55% were non-enzymatically converted to pEAβ42 after 24 h incubation time (Fig 3c). Although the incubation time was increased up to 3 days, an improvement of the E to pE conversion was not observable, most likely as a consequence of aggregation. Thus, we proved that the E3Q mutation facilitates and increases the yield of the final pEAβ significantly.

**Fig 4:**
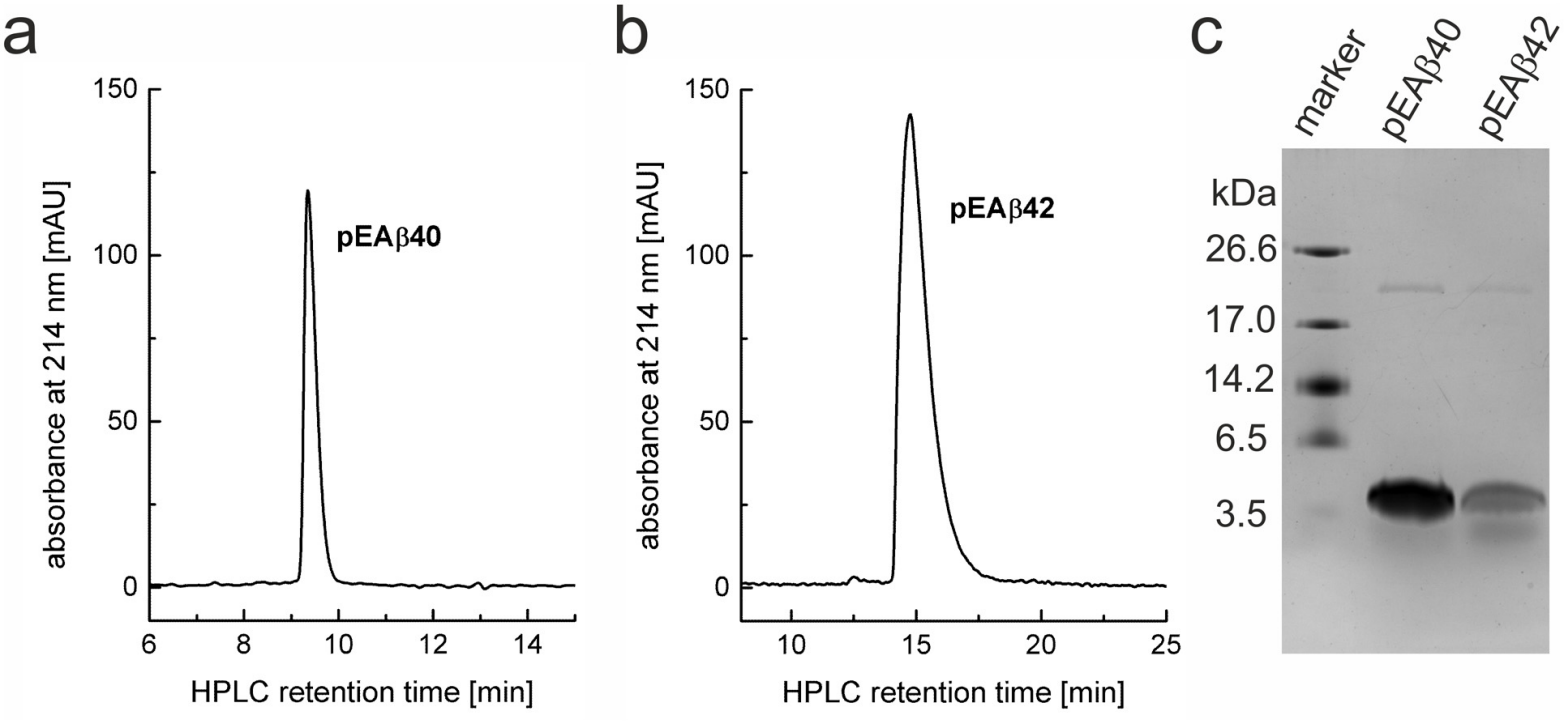
Analytics of final purified pEAβ. Analytical RP-HPLC of pEAβ40 (a) and pEAβ42 (b) after final purification and corresponding analysis of the proteins by Tris/Tricine-SDS-PAGE (c). The characteristic RP-HPLC retention times are approximately 9.5 min for pEAβ40 and 15 min for pEAβ42.

Since pEAβ precipitated completely during conversion the cleavage products were dissolved in 8 M GdmCl for preparative RP-HPLC purification. In this last purification step, it was possible to eliminate remaining impurities like non-pE-converted Aβ(E3Q-40/42). The purity of final pEAβ40 and pEAβ42 was checked by analytical RP-HPLC and by Tris/Tricine-SDS-PAGE [41] and determined to be more than 95% pure (Fig 4a–4c). Final yields of natural abundant pEAβ40 and pEAβ42 were 15 mg/l culture and 14 mg/l culture, respectively. Yields for isotope enriched pEAβ were approximately 2 mg/l culture as shown in Table 1.

### Biophysical characterization of pEAβ40 and pEAβ42

**Fig 5:**
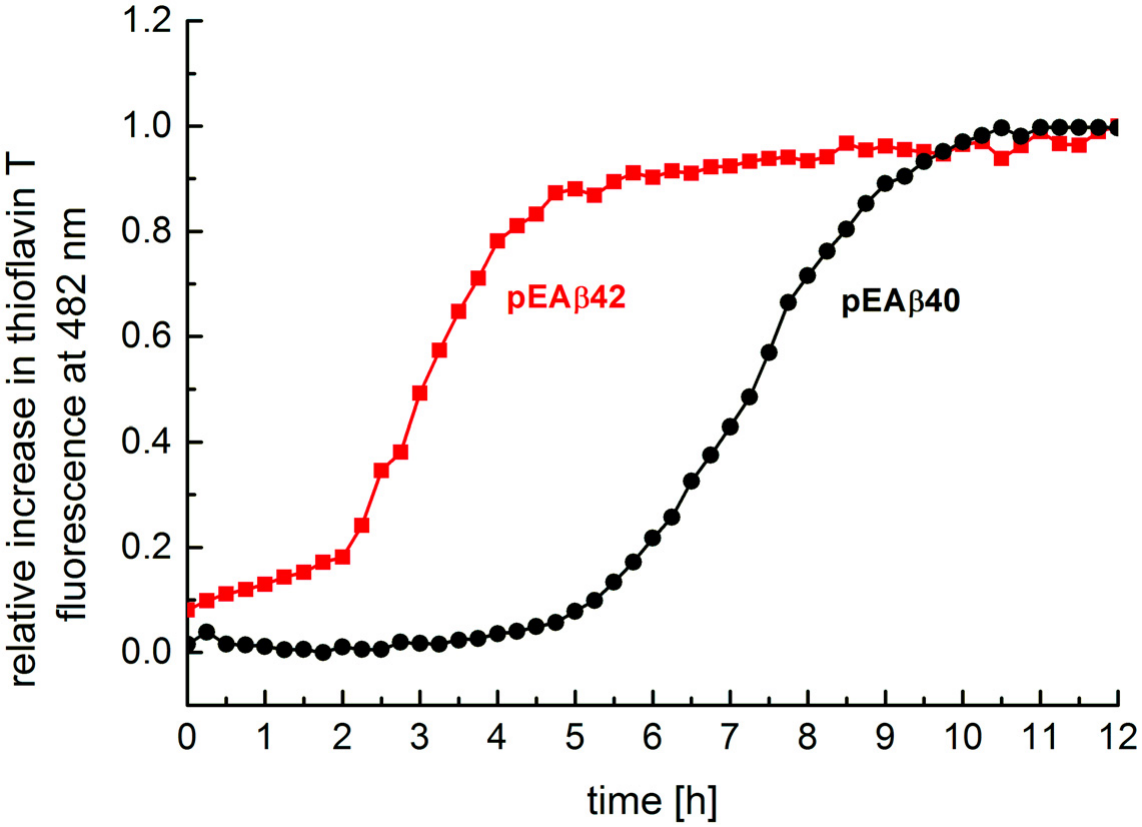
ThT assay of 10 μM recombinant pEAβ40 and pEAβ42. Experiments were performed in 10 mM sodium phosphate buffer pH 7.4 at 37°C. Binding of ThT (10 μM final concentration) to Aβ fibrils was determined by fluorescence at an extinction of 440 nm and emission at 492 nm.

Formation of amyloid-aggregates of various amyloidogenic proteins can be easily monitored by the commonly applied ThT assay [42]. Therefore, this assay was used to characterize the aggregation kinetics of recombinant pEAβ. Aggregation kinetics of 10 μM solutions of pEAβ40 and pEAβ42 were performed in near-physiological aqueous solution (sodium phosphate buffer, pH 7.4) at 37°C (Method G in S1 File). Both recombinant pE-modified Aβ peptides showed the typical properties of Aβ aggregation, i.e. a distinct lag phase, an elongation phase and a stationary phase over a 12 h incubation period (Fig 5). Compared to pEAβ40, pEAβ42 started to aggregate much faster, already before measurement was started, and reaches its stationary phase after 4 h. In contrast, at this time point (4 h) pEAβ40 just starts to overcome its lag phase monitored by ThT assay. Maximum ThT fluorescence intensity for pEAβ40 was observed after 10 h. The observed different aggregation kinetics of pEAβ40 and pEAβ42 can be explained by the fact, that the increased C-terminal length in pEAβ42 compared to pEAβ40 but also, in comparison to wild type Aβ(1-40/42) data, N-terminal deletions enhance aggregation [33, 43].

**Fig 6:**
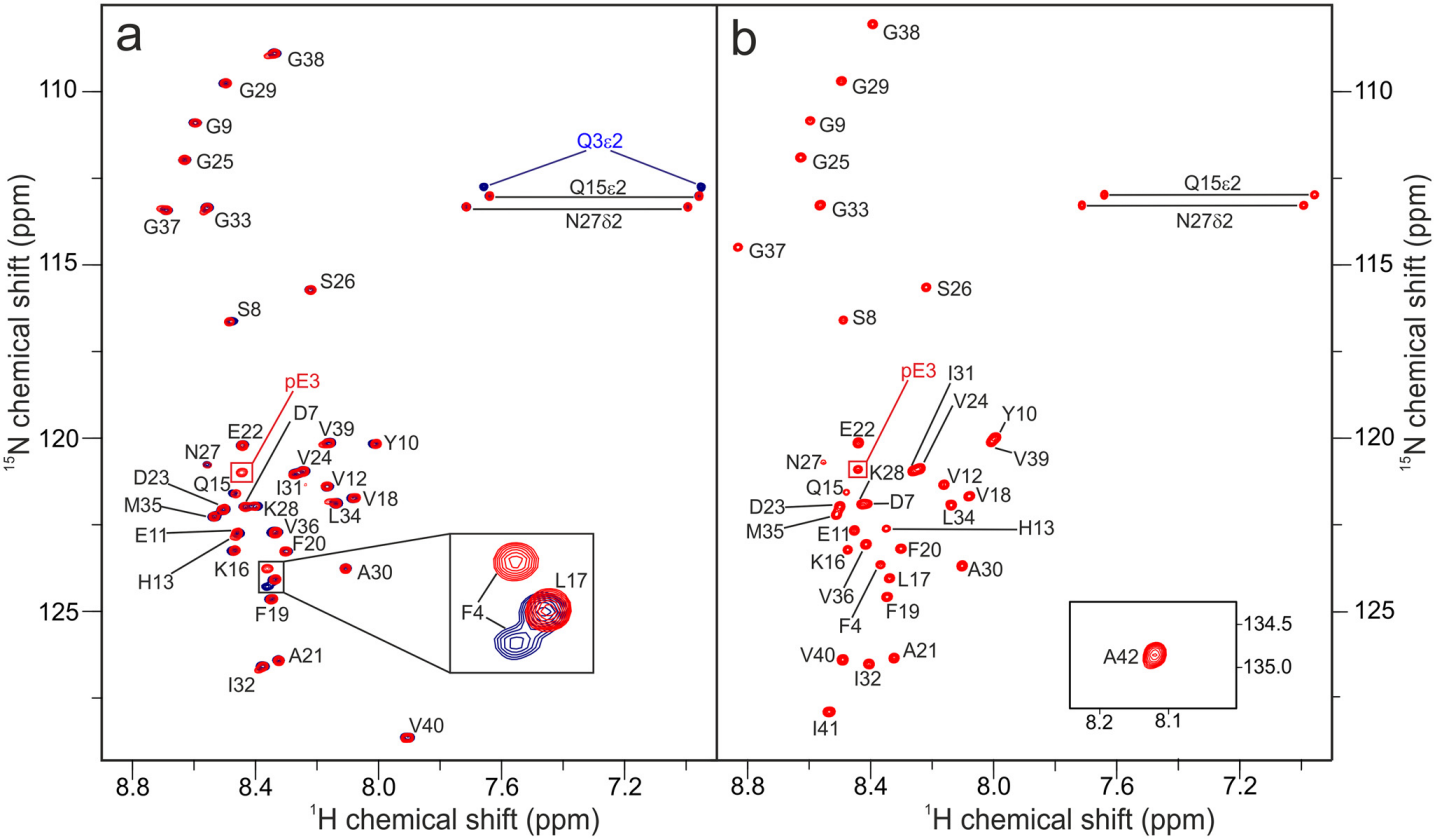
^1^H,^15^N-HSQC spectra of Aβ(E3Q-40) (blue) and pEAβ40 (red) (a) or of pEAβ42 (b). NMR spectra were recorded from 25 μM protein samples solved in 10 mM sodium phosphate buffer pH 7.4 at 5°C on a 600 MHz Bruker spectrometer. Note that in (a) “blue” signals derived from Aβ(E3Q-40) are overlaid with “red” signals from pEAβ40. Therefore “blue” signals are not visible, if identical in shift and intensity to “red” signals.

pEAβ40 and pEAβ42 were further analyzed by solution NMR spectroscopy. 2D and 3D NMR data were obtained at concentrations varying from 25 to 70 μM in aqueous solution at pH 7.4 and at 5°C (Method H in S1 File). No changes in chemical shifts could be detected within three days for pEAβ40 and pEAβ42. The NMR assignments were accomplished using BEST-TROSY HNCA+ experiments [44] for pEAβ40 and pEAβ42. Fig 6a displays an overlay of ^1^H,^15^N-HSQCs of pEAβ40 compared with the non-converted Aβ(E3Q-40). The loss of two signals from the γ-amino group due to deamination upon lactam ring formation as well as a shift of F4 and the appearance of a new signal of the pE3 peptide bond is the main difference. The spectra of pEAβ42 showed analogous results, the signals for the N-terminal amino group and the γ-amino group of Q3 are missing and a signal derived from the intramolecular pE3 lactam group appears (Fig 6b). However, compared with spectra of recombinant Aβ(1–40) and Aβ(1–42) under similar conditions published previously [45–47], the ^1^H,^15^N-HSQC NMR spectra of pEAβ40 and pEAβ42 differ slightly from Aβ(1-40/42). Although in both pEAβ species the N-terminal amino acid pE3 and the neighboring F4 are clearly visible, interestingly, R5 and H6 are missing not only in the pE modified peptides but also in the non-converted mutant E3Q, maybe due to the histidine-water proton exchange of H6 at neutral pH. Line-broadening and thus the disappearance of histidine signals were already described [48]. The intermediate acid-base proton exchange rate as well as the different tautomers of H6 might also affect the signal of the neighboring residue R5. D7 is shifted to lower frequency but from residue S8 on till the C-terminus, both NMR spectra for wild type Aβ(1-40/42) and for pEAβ40/42 are nearly identical.

## Conclusion

The described expression and purification system allows, for the first time, reproducible production of pEAβ in natural abundance and isotope-enriched in quantities up to 15 mg/l culture and overcomes the yield and costs limitations to perform reproducible biophysical studies. The purified pEAβ peptides (pEAβ40 and pEAβ42) showed elevated aggregation kinetics compared to Aβ(1–40) or Aβ(1–42), but, nonetheless, the monomeric states were suitable for biophysical studies at 5°C for at least three days. Moreover, it was possible to produce [*U*-^13^C,^15^N] pEAβ40 and pEAβ42 in high quality and quantity to perform high resolution NMR spectroscopy in solution state and to assign sequence specific signals of pEAβ40 and pEAβ42 under physiological conditions.

## Supporting Information

S1 File Method A. Cloning of recombinant plasmid encoding Aβ(E3Q-40/42) fusion protein. Method B. Expression and purification of Aβ fusion proteins. Method C. Cleavage of the fusion protein and purification of Aβ. Method D. Conversion to pEAβ40 and pEAβ42. Method E. pEAβ sample preparation. Method F. Mass spectrometry. Method G. Thioflavin-T assay. Method H. NMR spectroscopy.
(DOCX)
