# Supplementary material for: Purification and Characterization of Recombinant N-Terminally Pyroglutamate-Modified Amyloid-β Variants and Structural Analysis by Solution NMR Spectroscopy
Source: PLoS One. 2015 Oct 5;10(10):e0139710. doi: 10.1371/journal.pone.0139710 (PMC4593648; doi:10.1371/journal.pone.0139710)
Supplement: S1 File — Method A. Cloning of recombinant plasmid encoding Aβ(E3Q-40/42) fusion protein. Method B. Expression and purification of Aβ fusion proteins. Method C. Cleavage of the fusion protein and purification of Aβ. Method D. Conversion to pEAβ40 and pEAβ42. Method E. pEAβ sample preparation. Method F. Mass spectrometry. Method G. Thioflavin-T assay. Method H. NMR spectroscopy. (DOCX) [file pone.0139710.s001.docx]

# Supporting Information

**Purification and characterization of recombinant N-terminally pyroglutamate-modified amyloid-β variants and structural analysis by solution NMR spectroscopy**

*Christina Dammers^1^, Lothar Gremer^1,2^, Philipp Neudecker^1,2^, Melanie Schwarten^1^ and Dieter Willbold^1,2,*^*

*^1^Institute of Complex Systems (ICS-6) Structural Biochemistry, Forschungszentrum Jülich, 52425 Jülich, Germany*

*^2^Institut für Physikalische Biologie, Heinrich-Heine-Universität Düsseldorf, 40225 Düsseldorf, Germany*

*^*^*Corresponding author

E-mail: d.willbold@fz-juelich.de (DW)

## S1 Method A. Cloning of recombinant plasmid encoding Aβ(E3Q-40/42) fusion protein.

Cloning was based on a plasmid encoding wildtype Aβ(1-42) as a fusion protein with an N-terminal His_6_-tag, a solubilizing fusion partner (NANP)_19_ and a modified tobacco etch virus protease (TEV) recognition site, which was kindly provided by Finder, Glockshuber and coworkers [1]. Firstly, codons for D1 and A2 were deleted (Δ1/2) leading to the Aβ(3-42) construct. The following mutation of the encoding DNA sequence of E3 to Q (E3Q) leads to Aβ(E3Q-42) intended for increasing nonenzymatic conversion to pE. In addition, codons for the aa 41 and 42 (Δ41/42) were deleted to obtain the shortened variant ending with V40, i.e. Aβ(E3Q-40).

The oligonucleotides for deletion of codons for D1 and A2 and for codon mutation of E3 to Q were obtained (HPLC-grade) from Eurofins (Ebersberg, Germany) and the oligonucleotides to delete codons for I41 and A42 were obtained from BioTeZ (Berlin, Germany).

Δ1/2 forward: 5’-CTG AAA ACC TGT ATT TCC AGG AGT TCC GTC ATG ATT CAG-3’

Δ1/2 reverse: 5’-CTG AAT CAT GAC GGA ACT CCT GGA AAT ACA GGT TTT CAG-3’

E3Q forward: 5’-GAA AAC CTG TAT TTC CAG CAG TTC CGT CAT GAT TCA G-3’

E3Q reverse: 5’-GTG AAT CAT GAC GGA ACT GCT GGA AAT ACA GGT TTT C-3’

Δ41/42 forward: 5’-GGT GGG TGG TGT TGT CTA ATA GTA AAG CTT G-3’

Δ41/42 reverse: 5’-CAA GCT TTA CTA TTA GAC AAC ACC ACC CAC C-3’

Constructs for Δ1/2 and E3Q mutation were cloned using QuikChange Lightning Multi Site-Directed Mutagenesis Kit (Agilent, Böblingen, Germany). Amplification conditions were 30 cycles of 20 sec at 95 °C for denaturing, 30 sec at 55 °C for annealing and 2 min at 65 °C for elongation with *Pfu* ultra HF polymerase. Δ41/42 mutation of the E3Q construct was cloned with GeneArt® Site-Directed Mutagenesis PLUS System (Invitrogen Life Technology, Darmstadt, Germany) using 18 cycles of 50 sec at 95 °C for denaturing, 50 sec at 60 °C for annealing and 5 min at 68 °C for elongation with *Pfu* ultra HF polymerase. The amplified DNA was directly used for *Dpn*I digestion of the parental strain. *Escherichia coli* XL10 cells were transformed with the PCR product and grown clones were picked and sequenced by Seqlab (Göttingen, Germany).

## S1 Method B. Expression and purification of Aβ fusion proteins.

For expression of Aβ(E3Q-40/42) and wildtype Aβ(3-42) fusion proteins in natural abundance, a single colony of freshly transformed *E. coli* BL21 (DE3) pLysS cells was used to inoculated LB overnight starter-cultures (10 g/l tryptone, 5 g/l yeast extract and 5 g/l NaCl containing 100 mg/l ampicillin and 34 mg/l chloramphenicol). Main cultures of 1 l TB medium (12 g/l tryptone, 24 g/l yeast extract and 17 mM KH_2_PO_4_ and 72 mM K_2_HPO_4_ containing 100 mg/l ampicillin and 34 mg/l chloramphenicol) were inoculated with starter-cultures (1:100) and incubated at 37 °C until an OD_600 nm_ of 2.0 was reached. Protein expression was induced by adding isopropyl β-D-1-thiogalactopyranoside (IPTG) to a final concentration of 1 mM. The cells were incubated overnight at 30 °C and 160 rpm and then harvested by centrifugation (5,000 x g, 30 min). Cell pellets were resuspended in 5-10 ml lysis buffer (8 M guanidine hydrochloride (GdmCl)-NaOH, 300 mM NaCl, 30 mM Tris-HCl, 10 mM imidazole, pH 7.9) per gram wet weight, stirred for 2 h at 4 °C and then sonicated under ice cooling (3 x 1min, 50 % power, 5 mm micro-tip, Branson sonifier 250). Cell extract was clarified by centrifugation (50,000 x g, 45 min).

Uniformly [*U*-^15^N]- and [*U*-^13^C, ^15^N]-Aβ(E3Q-40/42) fusion proteins were expressed from cultures grown in M9 minimal media [2] with 1 g/l ^15^NH_4_Cl and 2.5 g/l unlabeled or [*U*-^13^C]-glucose being the sole nitrogen and carbon sources, respectively. Cultures were grown as described above for expression in natural abundance, with the difference that a starter-culture in non-isotope enriched M9 minimal media was prepared and used to inoculate 1 l main culture to an OD_600 nm_ of 0.1. The cells were then grown to an OD_600 nm_ of 1.2 and protein expression was performed overnight as described above.

The cell lysate was purified using immobilized metal affinity chromatography (IMAC) with 2 ml of Ni^2+^-NTA agarose (Qiagen, Hilden, Germany) per g of processed cells. The resin was equilibrated with lysis buffer and the cell extract was applied. After washing with 10 column volumes (CV) of lysis buffer, non-specifically bound protein was removed with 5 CV of lysis buffer including 20 mM imidazole. The fusion protein was eluted with 3 CV of 500 mM imidazole in lysis buffer and stored at -20 °C until further use. The eluate was checked by analytical reversed-phase high performance liquid chromatography (RP-HPLC) using a Zorbax SB-300 C8 column (5 µ, 4.6 mm x 250 mm, Agilent, Böblingen, Germany) connected to an Agilent 1200 Infinity system in 30 % acetonitrile (ACN) containing 0.1 % trifluoroacetic acid (TFA) at 80 °C and a flow-rate of 1 ml/min. Absorbance was measured at 214 nm. Data were recorded and analyzed with the program ChemStation (Agilent, Böblingen, Germany).

Further purification was performed via RP-HPLC using a semi-preparative Zorbax SB-300 C8 column (5 µ, 9.4 mm x 250 mm, Agilent, Böblingen, Germany) equilibrated with 20 % ACN/ 0.1 % TFA heated at 80 °C. IMAC purified fusion protein was directly loaded onto the column with a flow-rate of 4 ml/min. The column was washed for 12 min with 20 % ACN/ 0.1 % TFA and the fusion protein was then eluted with an isocratic step to 32 % ACN/ 0.1 % TFA and lyophilized.

## S1 Method C. Cleavage of the fusion protein and purification of Aβ.

The His_6_-tag and the solubilizing fusion partner (NANP)_19_ were cleaved from the fusion protein using TEV protease. Lyophilized fusion protein was resuspended in protease compatible buffer including 30 mM Tris-HCl, 1 mM EDTA, pH 8.0 and 3 mM dithiotreithol (DTT). 1 mg TEV protease per 8 mg fusion protein was added leading to a molar ratio around 0.06 equivalents relative to the fusion protein. Standard concentration of fusion protein was around 3-4 mg/ml. The cleavage reaction was performed at 4 °C for 20 h and was observed by analytical RP-HPLC isocratically with of 30 % ACN/ 0.1 % TFA at 80 °C. Since cleaved Aβ(E3Q-42) precipitated completely and Aβ(E3Q-40) partially during the reaction, the reaction mixture was centrifuged (16,000 x g, 20 min), the precipitate was dissolved in lysis buffer and both, supernatant and resuspended pellet, were reanalyzed by analytical RP-HPLC. Aβ containing fractions were purified on semi-preparative RP-HPLC (details see above). For Aβ(E3Q-42), the Zorbax SB300 C-8 column was equilibrated in 28 % ACN/ 0.1 % TFA and after sample loading washed for 12 min isocratically under equilibration conditions, then the target protein was eluted in an isocratic step with 35 % ACN/ 0.1 % TFA within 10 min. For Aβ(E3Q-40), the column was equilibrated with 30 % ACN/ 0.1 % TFA and protein was eluted isocratically with the same eluent. Pooled fractions containing Aβ(E3Q-40/42) were lyophilized and stored at -20 °C.

## S1 Method D. Conversion to pEAβ40 and pEAβ42.

Non-enzymatic conversion of Aβ(E3Q-40/42) to pEAß40 or pEAß42 was performed by incubation in mild acidic and elevated temperature condition. Therefore, lyophilized protein was dissolved in 30 mM sodium acetate, pH 3.5 (adjusted with acetic acid) to a concentration of 0.25 mg/ml and incubated at 45 °C with gentle stirring for 24 h. Conversion to pEAβ was analyzed by analytical RP-HPLC isocratically (see above) with 30 % ACN/ 0.1 % TFA at 80 °C and a flow-rate of 1 ml/min. Converted pEAβ40 and pEAβ42 precipitated completely during chemical reaction, therefore the reaction mixture was centrifuged (16,000 x g, 20 min) and the protein pellet was resuspended in lysis buffer. Purification of pEAβ was performed by semi-preparative RP-HPLC using the same conditions applied for the purification of unconverted Aβ(E3Q-40/42), respectively, and lyophilized.

## S1 Method E. pEAβ sample preparation.

Lyophilized pEAβ40 or pEAβ42 was dissolved in hexafluoroisopropanol (HFIP) and incubated at room temperature (RT) for at least three days to monomerize. Concentration was determined using a pEAβ40 calibration curve obtained by RP-HPLC. For this, 1 mg pEAβ40 was dissolved in 8 M GdmCl-NaOH pH 7.9 and diluted to final concentrations varying from 0 µM to 240 µM. The dilution series was applied on analytical Zorbax SB-300 C8 column in 30 % ACN/ 0.1 % TFA and eluted isocratically. A linear calibration curve was estimated due to the known concentration of pEAβ and the depended peak area detected at 214 nm. Based on this, all adjacent concentrations for recombinant pEAβ were calculated. Aliquots of 1 mg and specific aliquots for following experiments were prepared, lyophilized and stored at -20 °C until further use.

## S1 Method F. Mass spectrometry.

Mass spectrometry was performed using a high performance matrix-assisted laser desorption time of flight mass spectrometer (Voyager MALDI-TOF). Data were recorded between 1,000 and 15,000 m/z in positive-ion mode at the Leibniz-Institut für Umweltmedizinische Forschung (IUF, Düsseldorf, Germany).

## S1 Method G. Thioflavin-T assay.

Lyophilized pEAβ40 or pEAβ42 was dissolved in 10 mM H_3_PO_4_-NaOH, pH 7.4 and 10 µM ThT to a final concentration of 25 µM. Aggregation assays were performed using black non-binding 96-well plates (Sigma-Aldrich, Germany) at 37 °C. Wells were filled with samples to a total volume of 100 µl and each reaction was performed fivefold. Fluorescence was monitored with a microplate reader (PolarStar Optima, BMG, Offenburg, Germany) at excitation and emission wavelength of 440 and 492 nm, respectively, in bottom-reading mode every 15 min with 30 s shaking prior to reading. Fluorescence of the buffer excluding pEAβ was subtracted to correct for background.

## S1 Method H. NMR spectroscopy.

Lyophilized pEAβ was directly dissolved in 10 mM H_3_PO_4_-NaOH, pH 7.4 and 10 % D_2_O by vortexing. Sample concentrations varied between 25 and 50 µM. NMR spectra were acquired using a cryoprobe-equipped Bruker 600 MHz spectrometer at 5 °C in 5 mm standard NMR tubes. Chemical shift assignments were made using standard ^1^H,^15^N-HSQC and BEST-TROSY HNCA+ [3] experiments and spectra were processed with NMRPipe [4] and analyzed using CCPNmr Analysis [5]. ^1^H chemical shifts were referenced with respect to external DSS in D_2_O, ^13^C and ^15^N chemical shifts were referenced indirectly according to their gyromagnetic ratios [6, 7].

**References**

1. Finder VH, Vodopivec I, Nitsch RM, Glockshuber R. The recombinant amyloid-beta peptide Abeta1-42 aggregates faster and is more neurotoxic than synthetic Abeta1-42. Journal of molecular biology. 2010;396(1):9-18. Epub 2009/12/23. doi: 10.1016/j.jmb.2009.12.016. PubMed PMID: 20026079.

2. Maniatis T, Fritsch EF, Sambrook J. Molecular cloning : a laboratory manual. Cold Spring Harbor, N.Y.: Cold Spring Harbor Laboratory; 1982. x, 545 p. p.

3. Gil-Caballero S, Favier A, Brutscher B. HNCA+, HNCO+, and HNCACB+ experiments: improved performance by simultaneous detection of orthogonal coherence transfer pathways. Journal of biomolecular NMR. 2014;60(1):1-9. Epub 2014/07/25. doi: 10.1007/s10858-014-9847-x. PubMed PMID: 25056271.

4. Delaglio F, Grzesiek S, Vuister GW, Zhu G, Pfeifer J, Bax A. NMRPipe: a multidimensional spectral processing system based on UNIX pipes. Journal of biomolecular NMR. 1995;6(3):277-93. Epub 1995/11/01. PubMed PMID: 8520220.

5. Vranken WF, Boucher W, Stevens TJ, Fogh RH, Pajon A, Llinas M, et al. The CCPN data model for NMR spectroscopy: development of a software pipeline. Proteins. 2005;59(4):687-96. Epub 2005/04/09. doi: 10.1002/prot.20449. PubMed PMID: 15815974.

6. Wishart DS, Bigam CG, Yao J, Abildgaard F, Dyson HJ, Oldfield E, et al. 1H, 13C and 15N chemical shift referencing in biomolecular NMR. Journal of biomolecular NMR. 1995;6(2):135-40. Epub 1995/09/01. PubMed PMID: 8589602.

7. Markley JL, Bax A, Arata Y, Hilbers CW, Kaptein R, Sykes BD, et al. Recommendations for the presentation of NMR structures of proteins and nucleic acids--IUPAC-IUBMB-IUPAB Inter-Union Task Group on the standardization of data bases of protein and nucleic acid structures determined by NMR spectroscopy. European journal of biochemistry / FEBS. 1998;256(1):1-15. Epub 1998/09/24. PubMed PMID: 9746340.
